# Green Compressive Sampling Reconstruction in IoT Networks

**DOI:** 10.3390/s18082735

**Published:** 2018-08-20

**Authors:** Stefania Colonnese, Mauro Biagi, Tiziana Cattai, Roberto Cusani, Fabrizio De Vico Fallani, Gaetano Scarano

**Affiliations:** 1 DIET Department, University of Rome “La Sapienza”, 00184 Rome, Italy; mauro.biagi@uniroma1.it (M.B.); tiziana.cattai@uniroma1.it (T.C.); roberto.cusani@uniroma1.it (R.C.); gaetano.scarano@uniroma1.it (G.S.); 2 Inria, Aramis Project-Team, F-75013 Paris, France; fabrizio.devicofallani@gmail.com; 3 Institut du Cerveau et de la Moelle Épinière, ICM, Inserm U 1127, CNRS UMR 7225, Sorbonne Université, F-75013 Paris, France

**Keywords:** IoT network, energy efficiency, compressed sensing (CS), CS recovery, sensor networks

## Abstract

In this paper, we address the problem of green Compressed Sensing (CS) reconstruction within Internet of Things (IoT) networks, both in terms of computing architecture and reconstruction algorithms. The approach is novel since, unlike most of the literature dealing with energy efficient gathering of the CS measurements, we focus on the energy efficiency of the signal reconstruction stage given the CS measurements. As a first novel contribution, we present an analysis of the energy consumption within the IoT network under two computing architectures. In the first one, reconstruction takes place within the IoT network and the reconstructed data are encoded and transmitted out of the IoT network; in the second one, all the CS measurements are forwarded to off-network devices for reconstruction and storage, i.e., reconstruction is off-loaded. Our analysis shows that the two architectures significantly differ in terms of consumed energy, and it outlines a theoretically motivated criterion to select a green CS reconstruction computing architecture. Specifically, we present a suitable decision function to determine which architecture outperforms the other in terms of energy efficiency. The presented decision function depends on a few IoT network features, such as the network size, the sink connectivity, and other systems’ parameters. As a second novel contribution, we show how to overcome classical performance comparison of different CS reconstruction algorithms usually carried out w.r.t. the achieved accuracy. Specifically, we consider the consumed energy and analyze the energy vs. accuracy trade-off. The herein presented approach, jointly considering signal processing and IoT network issues, is a relevant contribution for designing green compressive sampling architectures in IoT networks.

## 1. Introduction

Compressive sampling (CS) is a wide area of studies concerning the representation of a sparse *N*-dimensional signal from a limited set of M<N random projections. A promising CS application area is that of IoT networks for environmental monitoring, i.e., IoT networks aimed at capturing time varying fields representing physical data, e.g., temperature, chemical composition and so on.

In a nutshell, monitoring consists of the following stages: (S1) periodically acquiring *M* CS measurements at a temporal pace tuned to the field temporal coherence, (S2) gathering them at a specific sink node, and (S3) reconstructing the field on a bidimensional spatial grid. Thereby, we identify different components for the energy consumed by the IoT network, namely:

- CS measurement acquisition and gathering energy $E_{S_{1}}$ and $E_{S_{2}}$, consumed during the stages S1 and S2;
- CS reconstruction energy $E_{S_{3}}$, corresponding to stage S3.

In IoT networks, minimizing the overall consumed energy $E_{IoT}=E_{S1}+E_{S2}+E_{S3}$ is a key design goal affecting both the network life time and the maintenance costs [1]. Several works in the literature focus on the energy consumption of the first and second stage (S1) and (S2), namely sensing and data gathering, including the energy spent at the sink during the data reception stage [2,3,4]. As for the sensing, the CS matrix may be selected according to different criteria (see [5,6] for comprehensive surveys) and lead to differentiated performances [7]. When implemented in an IoT network, the structure of the sensing matrix and the gathering of the CS measurements at the sink directly affect the energy components $E_{S1},E_{S2}$. An example is found in [8], where the random sensing matrix whose statistical properties where presented in [9] is adopted. Therein, the author show that suitable sensing matrix selection may lead to significant energy savings in the CS measurements acquisition/gathering stage as discussed in [10] and, for the case of multimedia acquisition, in [11,12]. Thereby, the energy for transmitting data from sensors to the sink varies according to the selected data-gathering strategy as discussed above. Once the CS measurements are available at the sink, signal reconstruction is purposed at the cost of further energy consumption. Only few pioneering works focus on the energy spent in the CS reconstruction stage S3). In [13], the authors analyze the impact of lowering power consumption in sensor networks by rakeness-based CS on the reconstruction performance, and they compare on-board power consumption for different reconstruction algorithms. The power consumption dependence on r parameters such as the number of measurements and the computation time is analyzed. In [14], the authors consider the quality–energy trade-off of a sensors network using where different hubs are considered for intermediate data aggregation; the compression capability of CS with a highly simplified digital hardware is investigated in [15].

Herein, we carry out the analysis of the CS reconstruction energy $E_{S3}$, focusing on the impact of the computational architecture on the IoT network energy consumption. When CS is considered in a IoT network, CS reconstruction can be either (i) offloaded for computation outside the network or (ii) performed within the network itself. These two methods are expected to differ in terms of energy consumption; therefore, some relevant questions arise:

- Which are the system parameters that affect the IoT networks’ energy consumption in these two cases?
- Under which conditions offloading CS reconstruction is more energy efficient than performing in-network CS reconstruction?
- Are there criteria to select the CS reconstruction algorithm not only in terms of achieved accuracy but also in terms of energy vs. accuracy trade-off?

This paper answers to these questions. Specifically, herein, we analyze the network energy consumption for the CS reconstruction stage in two computing architectures:

- in the first one, referred to as *off-network reconstruction*, all the CS measurements are forwarded to off-network devices for reconstruction and storage;
- in the second one, named *in-network reconstruction*, the reconstruction takes place within the IoT network itself, the reconstructed data are then encoded and eventually forwarded out of the IoT network.

Our analysis of the cost of in-network and off-network reconstruction highlights the trade-off between energy spent either in processing or in transmission processes, which is faced in mobile computing as well [16]. The result of the analysis provides insight on aforementioned system design issues.

The structure of the paper is as follows. In Section 2, we outline the model underlying CS in IoT networks. In Section 3, we provide an analysis of the energy cost of both in-network or off-network CS reconstruction, and, in Section 5, we address two specific applications of the proposed analysis and present numerical results for both cases under realistic settings. Section 7 concludes the paper.

## 2. CS in IoT Networks

The goal of CS in field monitoring by IoT networks is to reconstruct, at a temporal pace of $T_{s}$ [s], a snapshot of the values assumed by the monitored field in *N* points. Typically, the *N* points are located on a regular grid, and CS reconstruction recovers a bidimensional sequence $x_{mn}$, given a number *M* of CS measurements significantly lower than *N*. We denote by x the vector representing $x_{mn}$ in lexicographic form.

Underlying physical constraints typically limit the degrees of freedom of the sensed field. Thereby, x is *N*-dimensional, but, in most cases, it is *K* sparse under a transformation identified by a transform basis matrix T, i.e., x can be expressed as

x=Tα,

where the transform coefficients’ vector α has exactly *K* non zero coefficients.

The *M*-dimensional CS measurements vector y is acquired by data gathering procedures taking place within the network, and it is modeled as

(1) $$y=\tilde{\Phi }x+n=\tilde{\Phi }T\alpha +n=\Phi \alpha +n,$$

where $\tilde{\Phi }$ denotes a suitable M×N random sensing matrix meeting the conditions for perfect reconstruction of x given y, $\Phi =\tilde{\Phi }\;T$ and n is the acquisition noise.

The sparse coefficients α are jointly distributed in a way depending on the adopted transformation T (see, for instance, [17,18,19]), and reconstruction algorithms may leverage signal priors to further reduce the dimensionality of the solution subspace.

**Figure 1:**
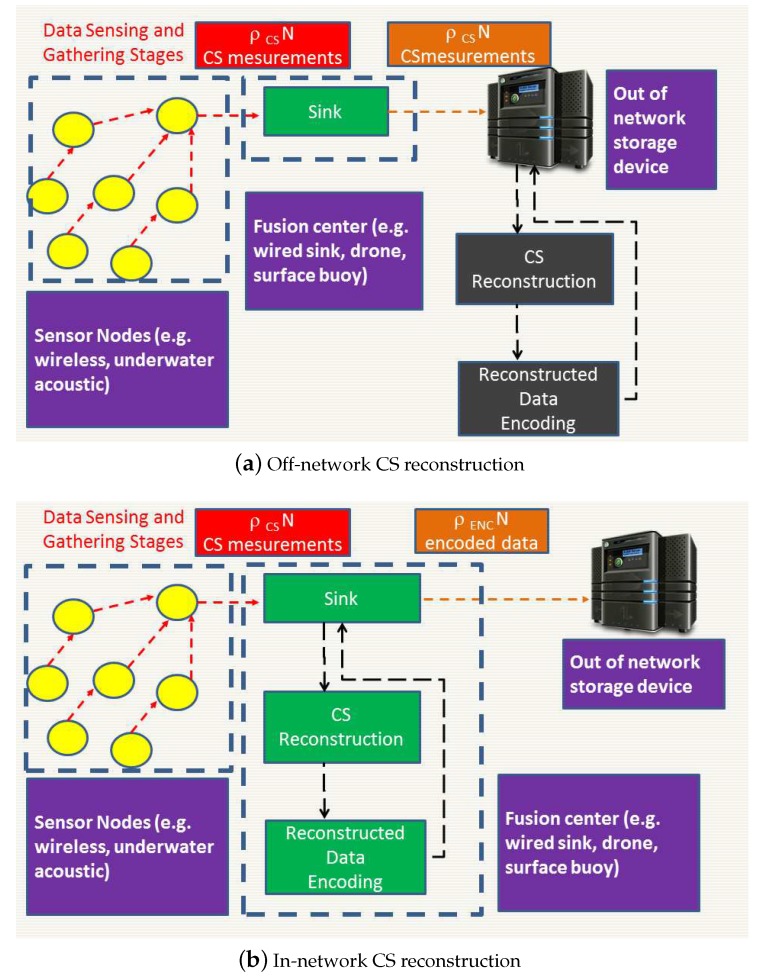
Computing architectures for IoT CS reconstruction.

When CS is adopted in IoT networks, different stages contribute to the overall energy cost. Firstly, the field is sensed every $T_{s}$ seconds. Secondly, data are transmitted towards a node called sink with higher capabilities such as transmission bandwidth or energy resources. The energy cost varies with the number of collected measurements, the network architecture and the access protocols. Thirdly, CS reconstruction is performed. Herein, we focus on CS reconstruction, and we develop our analysis for two alternative computational architectures, illustrated in the upper and lower part of Figure 1.

According to the first computational architecture, which we refer to as off-network reconstruction, the sink off-loads the reconstruction out of the IoT network. Thereby, it transmits all the CS measurements and leaves the task of CS reconstruction to off-network devices. This is schematically illustrated in the upper part of Figure 1; we recognize that, after the sensing stage, during the data gathering stage the CS measurements are collected at the sink node. During the reconstruction stage, the received data are just forwarded out of the network for proper computation.

In the second strategy, which we refer to as in-network reconstruction, the reconstruction firstly takes place within the IoT network itself, the reconstructed data are then compressed using appropriate coding and finally the encoded bitstream is forwarded out of the IoT network. This computing architecture is schematically illustrated in the lower part of Figure 1. Therein, we recognize that data are sensed within the IoT network and gathered at the sink, just as mentioned above for off-network reconstruction. As for the reconstruction stage, it takes place within the IoT network where CS measurements are processed for reconstruction. After this stage, the reconstructed data are encoded for transmission and finally forwarded to the external storage system for application dependent processing.

The first computational architecture appears more immediate, but it is not guaranteed to be the most energy efficient. Herein, we evaluate the network energy consumption implied by both off-network and in-network reconstruction. We show that in both cases the consumed energy depends on the IoT network features. Thereafter, we identify the relevant system parameters and analyze the conditions under which the architectures outperform each other in terms of energy efficiency. The notation adopted in the following analysis is summarized in Table 1.

## 3. Relevant Parameters for Energy Efficiency Analysis of CS Reconstruction

Let us first analyze the case of off-network reconstruction. In this scheme, the computation of the signal given the CS measurements is off-loaded. Thereby, the energy $E_{S3}$ spent within the network equals to the energy $E_{\mathrm{off}-\mathrm{net}}$ required for transmitting the CS measurements acquired during an observation time *T* at a temporal sampling pace $T_{s}$; we denote this energy by $E_{CS\;-\;DTX}$. We observe that $E_{CS\;-\;DTX}$ is proportional to the number of CS measurements *M* as well as to the number of bits *L* required to represent each CS sample:

(2) $$E_{\mathrm{off}-\mathrm{net}}=E_{CS\;-\;DTX}=T/T_{s}MLE_{b},$$

where we denote by $E_{b}$ the average energy required for the transmission of one bit. It is worth noting that the parameter $E_{b}$ summarizes the energy consumption of the physical and link layer procedures in terms of average energy employed for transmitting one bit at the desired quality, and therefore varies widely from a transmission technology and physical channel to another. In turn, we observe that the number *M* of CS measurements required for accurate reconstruction of a *N*-size image can be expressed a fraction of *N*; the ratio $\rho_{CS}=M/N$ represents the CS compression efficiency and it depends on the signal sparsity and on the sensing matrix. Thus, we obtain

(3) $$E_{\mathrm{off}-\mathrm{net}}=T/T_{s}\cdot L\rho_{CS}NE_{b}.$$

In case of in-network reconstruction, different energy draining stages can be identified. Specifically, the following tasks are to be considered:

(T1) reconstruction from CS measurements, consuming $E_{REC}$,
(T2) reconstructed data encoding, consuming $E_{ENC}$,
(T3) data transmission, consuming $E_{REC\;-\;DTX}$.

With these positions, the overall energy consumption $E_{S3}$ during the observation time interval *T* over which the field is monitored, equals:

(4) $$E_{\mathrm{in}-\mathrm{net}}=T/Ts\left(E_{REC}+E_{ENC}+E_{REC\;-\;DTX}\right).$$

As far as the reconstruction energy term $E_{REC}$ is concerned, we observe that, according to [20], the number of elementary processor operations $n_{p}$ required by the reconstruction algorithm is upper-bounded by a value that is proportional to both the number *M* of the CS measurements and the image size *N*, i.e., $n_{p}\leq \kappa_{1}\;M$. The empirical proportionality factor $\kappa_{1}$ depends on the implemented reconstruction algorithms; for instance, in [20], the authors show that $\kappa_{1}\approx 1$ for the therein proposed Compressive Sampling Matched Pursuit (CoSaMP) algorithm. Thereby, denoting by $E_{p}$ the energy of the elementary processor operation, we can express the energy required for reconstruction as

(5) $$E_{REC}=n_{p}\;E_{p}=\kappa_{1}MNE_{p}=\kappa_{1}\rho_{CS}N^{2}E_{p}.$$

As far as the encoding stage is concerned, the energy $E_{ENC}$ is linearly related on the overall encoding cost which depends on the number of coding blocks and, in turn, on the image size *N* [21]. The overall energy spent to encode the reconstructed data can then be expressed as

$$E_{ENC}=\kappa_{2}NE_{p}.$$

The empirical constant $\kappa_{2}$ depends on the actual encoding procedure and it has been analyzed for different codecs. For instance, it has been shown that $\kappa_{2}\approx 1$ for H.263 [21] and $\kappa_{2}\approx 2÷3$ for H.264 video coding [22].

Finally, we consider the energy $E_{REC\;-\;DTX}$ required for transmitting the reconstructed and encoded data to the external storage system, which is as well proportional to the number of encoded bits and to the per-bit transmission energy $E_{b}$. Thereby, if we denote by $\rho_{enc}$ [bpp] the number of bits per pixel characterizing the encoder efficiency, the number of encoded bits needed to transmit one snapshot of the field is given by $\rho_{enc}N$. Then, we can express $E_{REC\;-\;DTX}$ as:

$$E_{REC\;-\;DTX}=\rho_{enc}NE_{b}.$$

A few remarks are in order. Firstly, it is worth noting that we assume from now on that the channel quality specifications are the same in the in-network and off-network schemes, and $E_{b}$ is the same in the two cases. This assumption is fair since both CS measurements and modern video encoders respectively have intrinsic error resilience properties as well as efficient error resilience tools; therefore, both techniques can deal with comparable transmission error rates.

As for the impact of the quantization of the CS measurements employed in the case, we assume that the number of bits per sample *L* selected for transmission assures ideal reconstruction. Still, we observe that quantization may affect, to a larger extent, the accuracy of CS reconstruction in the case of off-network reconstruction. In fact, in the in-network reconstruction scheme, the CS reconstruction algorithm processes the actual CS measurements, typically quantized just once while being gathered through the network. In case of off-network reconstruction, the CS reconstruction algorithm is fed by CS measurements that may be quantized twice, i.e., both in the gathering stage and for transmission out of the IoT network. Thereby, both of the schemes are affected by a non-ideal, lossy representation. We disregard these effects since they are of second order w.r.t. typical CS reconstruction errors.

It is worth asking how the structure of the sensing matrix, the sparsity of the signal, and even the acquisition noise affect the reconstruction energy. We observe that these application layer design choices straightforwardly influence the number *M* of measurements needed to achieve the desired reconstruction accuracy. Thereby, their effect on the energy spent in reconstruction is accounted for by the parameter $\rho_{CS}$, whose relevance is widely discussed in the following analysis.

Finally, for completeness sake, let us mention that also the bandwidths $B_{\mathrm{in}-\mathrm{net}},B_{\mathrm{off}-\mathrm{net}}$ employed in the in-network and off-network approaches are different, reflecting the circumstance that either the quantized CS measurements or the encoded reconstructed signal are transmitted. The ratio between the bandwidths is expressed as:

$$\frac{B_{\mathrm{in}-\mathrm{net}}}{B_{\mathrm{off}-\mathrm{net}}}=\frac{\rho_{enc}}{L\rho_{CS}}.$$

## 4. Comparison of In-Network and Off-Network Reconstruction Schemes

To compare the energy efficiency of in-network versus off-network reconstruction, we consider the ratio of the energy costs of the two strategies:

(6) $$R_{in}=\frac{E_{\mathrm{in}-\mathrm{net}}}{E_{\mathrm{off}-\mathrm{net}}}=\frac{\overset{REC}{\overset{︷}{\kappa_{1}\rho_{CS}NE_{p}}}+\overset{ENC}{\overset{︷}{\kappa_{2}E_{p}}}+\overset{DTX}{\overset{︷}{\rho_{enc}E_{b}}}}{\underset{CS\;-\;DTX}{\underset{︸}{L\rho_{CS}E_{b}}}},$$

and we introduce the decision function

(7) $$D_{in}=1\;\Leftrightarrow R_{in}<1,$$

which equals to one when and only when in-network reconstruction is more energy efficient than off-network reconstruction. From Equation (6), we recognize that $D_{in}$ is a multivariate function that varies with a bunch of parameters, among which we recognize application layer parameters such as (i) the image size; (ii) the CS coding and image coding efficiency, and, even most important, technological features such as the (iii) energy required per bit transmission and the (iv) energy per processing operation. In the following, we show how the analysis summarized in Equation (6) applies to the actual parameter choices.

To elaborate, let us explicitly analyze the relation between the in-network reconstruction decision function $D_{in}$ and

- the system related energy parameters $E_{b}$ and $E_{p}$;
- the application layer parameters, specifically the field size *N*, the compression efficiency of CS $\rho_{CS}$ and that of the encoding stage $\rho_{enc}$.

First, we analyze under which constraint on the system related energy parameters $E_{b}$ and $E_{p}$ in-network reconstruction is more energy efficient than off-network reconstruction. After applying some simple algebra to Equation (6), we obtain that $D_{in}=1$ for value of the ratio $E_{b}/E_{p}\left(\mathrm{dB}\right)$ satisfying

(8) $$\frac{E_{b}}{E_{p}}\geq \frac{\kappa_{1}\rho_{CS}N+\kappa_{2}}{L\rho_{CS}-\rho_{ENC}}.$$

**Figure 2:**
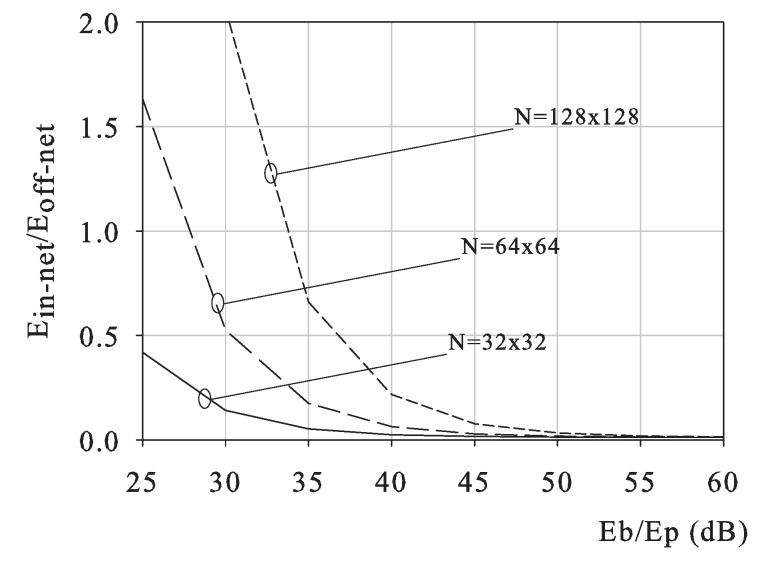
$E_{\mathrm{in}-\mathrm{net}}/E_{\mathrm{off}-\mathrm{net}}$ versus $E_{b}/E_{p}$, $L=8,\;\rho_{CS}=0.2,\;\rho_{enc}=0.02$.

The above reported analytical result is observed in Figure 2, where we plot the ratio $E_{\mathrm{in}-\mathrm{net}}/E_{\mathrm{off}-\mathrm{net}}$ versus the ratio $E_{b}/E_{p}\left(\mathrm{dB}\right)$ for different values of *N*, namely N=32×32, N=128×128.

For the sake of completeness and reproducibility, let us observe that the results in Figure 2 have been obtained by setting $\kappa_{1}$, as in [20] ($\kappa_{1}\approx 1$), and computing $\kappa_{2}$ following the video encoding energy as in [21]. Therein, the authors show that, for the H.263 case, the overall video encoding energy $E_{H.263}$ is up to 140% of the transform coding energy cost $E_{TC}$, which in turn depends on the block size (8×8=64 pixels for H.263 encoding), namely $E_{TC}=2\times 64\log\left(2^{3}\right)N/64E_{p}$. Resorting to the same computation for the case under concern, we come up to $\kappa_{2}\approx 6\log\left(2\right)\cdot 1.4\approx 1$. Still, the main trends herein observed are maintained for small percentage variations of $\kappa_{1},\kappa_{2}$.

In Figure 2, we observe that the minimum $E-b/E_{p}$ such that $R_{in}$ varies with the networks size *N*. For a small N=32×32 network, $R_{in}$ is less than one for a wide range of the value $E_{b}/E_{p}$, whereas, for N=64×64 and 128×128 networks, the minimum $E_{b}/E_{p}$ such that $R_{in}$ increases to 28 and 32 dB, respectively. The reason why this occurs is that for increasing network size the energy spent in processing increases faster than the transmission energy, and in-network reconstruction becomes less efficient. Whether the in-network scheme saves energy w.r.t. the off-network one or not depends on the relative cost of data transmission and processing, represented by $E_{b}/E_{p}$. Thereby, as the network size increases, the threshold value of $E_{b}/E_{p}$ increases as well, as described by Equation (8).

Next, we extend the analysis to the effect of $\rho_{CS}/\rho_{enc}$ on the energy efficiency of the two strategies. In particular, we show by algebraic manipulation of Equation (6) that the difference of compression efficiency between the two schemes affects the overall energy consumption. In addition, the extent of the impact depends on the ratio $E_{b}/E_{p}$ as well, i.e., on how much the energy cost of transmission exceeds that of processing.

**Figure 3:**
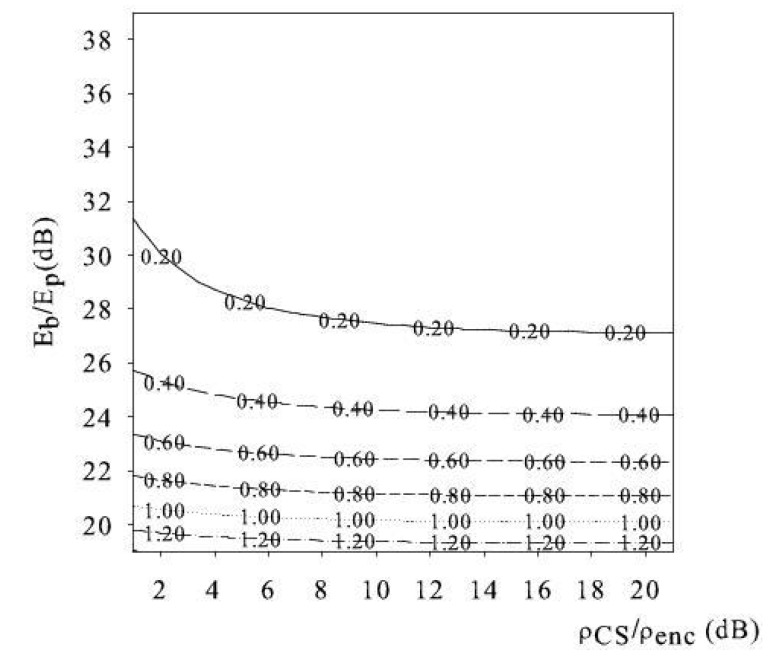
Ratio $R_{in}=E_{\mathrm{in}-\mathrm{net}}/E_{\mathrm{off}-\mathrm{net}}$ versus the ratio $\rho_{CS}/\rho_{enc}$ and $E_{b}/E_{p}$ (N=32×32, *L* = 8).

**Figure 4:**
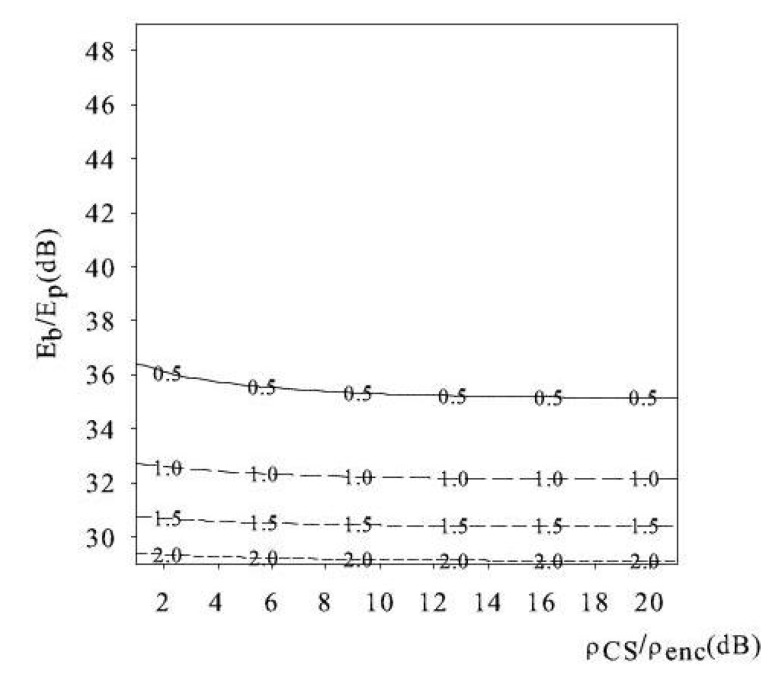
Ratio $E_{\mathrm{in}-\mathrm{net}}/E_{\mathrm{off}-\mathrm{net}}$ versus the ratio $\rho_{CS}/\rho_{enc}$ and $E_{b}/E_{p}$ , N=128×128, *L* = 8).

To illustrate this behavior, in Figure 3 and Figure 4, we show the level curves of the ratio $R_{in}=E_{\mathrm{in}-\mathrm{net}}/E_{\mathrm{off}-\mathrm{net}}$ vs. the pair $\left(E_{b}/E_{p},\rho_{CS}/\rho_{enc}\right)$, for N=32×32, N=128×128, respectively. The level curves present a small slope, indicating that the variation of the ratio $R_{in}$ faster w.r.t. to the variation of the ratio $E_{b}/E_{p}\left(\mathrm{dB}\right)$ than of the ratio $\rho_{CS}/\rho_{enc}$. Thereby, $R_{in}$ is robust to slightly different choices of the number of CS measurements and/or of the sensing matrices, which affect $\rho_{CS}$. In addition, $R_{in}$ is robust to fluctuation of the coding efficiency, affecting $\rho_{enc}$. From observations of Figure 3 and Figure 4, we recognize that the ratio $R_{in}=E_{\mathrm{in}-\mathrm{net}}/E_{\mathrm{off}-\mathrm{net}}$ is below 1 for wide ranges of $E_{b}/E_{p}\left(\mathrm{dB}\right)$ and $\rho_{CS}/\rho_{enc}$. In other words, there is a wide region of system parameters under which in-network reconstruction is preferable, i.e., $D_{in}=1$.

In the limit case when $\rho_{CS}/\rho_{enc}\gg 1$, the ratio $R_{in}$ tends to a horizontal asymptote. The reason why this occurs is that, for this condition, the dominant term of the in-network energy consumption is the reconstruction energy $E_{REC}$, and

$$\frac{E_{\mathrm{in}-\mathrm{net}}}{E_{\mathrm{off}-\mathrm{net}}}\approx \frac{\kappa_{1}}{L}NE_{p}/E_{b}.$$

Thereby, the cost of in-network reconstruction when $\rho_{CS}/\rho_{enc}\gg 1$ is dominated by the processing energy component and varies with the field size *N*.

It is worth asking if this processing energy dominated condition is achievable in practical cases. To see this, let us recall that still image coding techniques employ 0.1 bpp [23], and video coding techniques achieve much lower rates, typically $\rho_{enc}\in \left(0.01-0.1\right)$. On the other hand, the ratio $\rho_{CS}=M/N$ is usually set to 0.2, and it may increase up to 0.7 in peculiar cases, as in Random Sensing of spatially localized images [2,8,24]. Thereby, in many practical cases, we obtain $D_{in}=1$, i.e., in-network is the most energy efficient solution just when $N<LE_{b}/E_{p}$

Finally, it is worth noticing that, for values of $E_{b}$ in $E_{p}<E_{b}<NE_{p}$, we can also use $R_{in}\approx \left(\kappa_{1}\rho_{CS}N+\rho_{enc}E_{b}/E_{p}\right)/\left(L\rho_{CS}Eb/Ep\right)$. Finally, when $E_{b}>>NE_{p}$, Equation (6) boils down to $R_{in}=\rho_{enc}/(L\rho_{CS}$ (i.e., the most energy efficient scheme is the one associated with the most efficient compression algorithm). The condition $E_{b}>>NE_{p}$ is much more demanding than Eb>>Ep, especially when the network size increases .

Under the same settings, side beneficial effects of in-network reconstruction are expected in terms of saved bandwidth. In Table 2, we report the ratio between $B_{\mathrm{in}-\mathrm{net}}$ and $B_{\mathrm{off}-\mathrm{net}}$ for different values of the ratio $\rho_{CS}/\rho_{enc}$. We observe that $B_{\mathrm{in}-\mathrm{net}}$ is typically lower than $B_{\mathrm{off}-\mathrm{net}}$.

In the following, we apply the above analysis to selection of the most efficient computing architecture for the IoT network under concern and performance comparison of CS reconstruction algorithms in terms of energy vs. accuracy trade-off.

## 5. Selection of Energy Efficient Computing Architecture for CS Reconstruction

Given the values of the main system parameters that impact the energy efficiency of the computing architecture have been identified, the analysis allows for selecting the computing architecture more suited to the case under concern. Specifically, according to Equation (6), there exists a maximum value $N_{in}$ of the number of samples to be reconstructed *N* such that in-network reconstruction is preferable to off-network reconstruction from an energy efficiency point of view, i.e., $D_{in}=1$ for $N\leq N_{in}$. $N_{in}$ can be computed as:

(9) $$N_{in}=\frac{\left(L\rho_{CS}-\rho_{enc}\right)\frac{E_{b}}{E_{p}}-\kappa_{2}}{\kappa_{1}\rho_{CS}}.$$

In Table 3, we report the values of $N_{in}$ in three reference scenarios, namely a wireless IoT network scenario and two underwater acoustic network, respectively using IEEE 802.15.4 compliant radio transceivers [25], acoustic modem as those described in [26] and acoustic modem as those described in [27,28] ($@\rho_{CS}=0.25,\rho_{enc}=0.25$). We recognize that, in the wireless scenario, the bit transmission energy $E_{b}$ corresponds to a few hundred $E_{p}$, and it can further increase at higher transmission rates or in harsh channel conditions. On the other hand, in an underwater network, the ratio $E_{b}/E_{p}$ may be as high as $10^{7}$. Although the value of $E_{b}$ significantly varies with the modulation technique, the device-related processing energy $E_{p}$ is a small fraction of the energy spent at the transmission interface [29]. Thereby, in IoT networks, based on wireless sensor technology in-network reconstruction is to be preferred in the case of limited field size N>800, whereas, in underwater acoustic networks, where the energy consumption due to transmission is very high, in-network reconstruction is preferable also for huge fields.

### Key Take-Aways

In principle, the energy design of the reconstruction stage should be carried out jointly with the sensing and gathering stage. This notwithstanding, we observe that the choice of the sensing matrix affects the energy spent during the data sensing and gathering stages. As for the reconstruction stage, the sensing matrix and the signal sparsity affect the system through of the parameter $\rho_{CS}$, specifying the fraction of measurements needed for reconstruction. Herein, it has been shown that the energy spent during the reconstruction stage weakly depends on this parameter. Thereby, given $\rho_{CS}$, the computation of the energy spent in the reconstruction stage can be carried out independently from the application layer details of the sensing and gathering stage.

**Figure 5:**
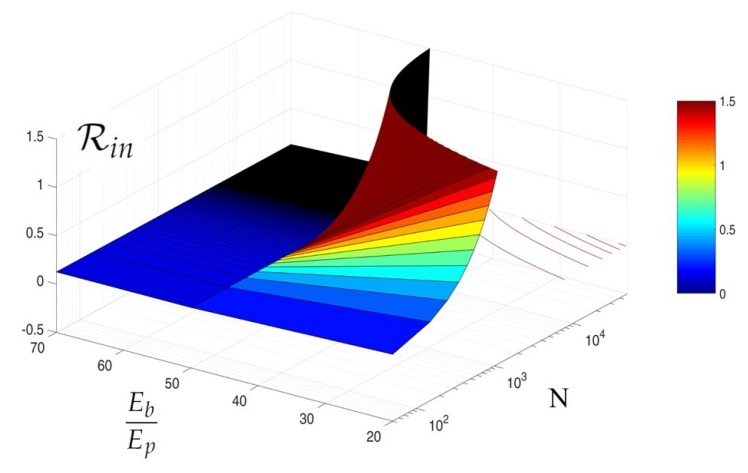
$R_{in}$ versus $E_{b}/E_{p},N$, $L=8,\;\rho_{CS}=0.25,\;\rho_{enc}=0.25$.

As for the joint dependence on $E_{b}/E_{p}$, and *N*, we observe that the most appropriate architecture for the scenario under concern jointly depends on these two parameters. In Figure 5, we plot $R_{in}$ versus $E_{b}/E_{p},N$, $L=8,\;\rho_{CS}=0.25,\;\rho_{enc}=0.25$. In-network reconstruction is preferable when $R_{in}<1$ There is clearly a combination of network size and energy required for transmission versus processing energy such that off-network reconstruction is preferable, namely for large network using low-energy transmission.

**Figure 6:**
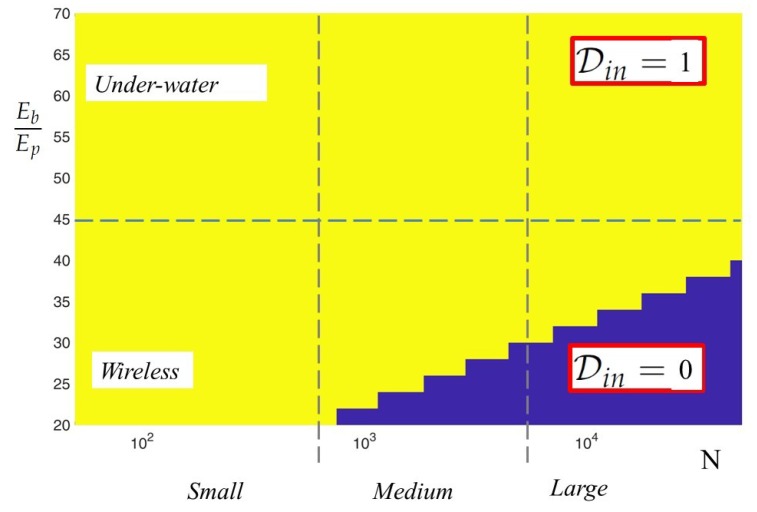
$D_{in}$ versus $E_{b}/E_{p},N$, $L=8,\;\rho_{CS}=0.25,\;\rho_{enc}=0.025$.

For concreteness’ sake, in Figure 6, we also plot $D_{in}$ versus $E_{b}/E_{p},N$, and highlight regions of the plane $E_{b}/E_{p},N$, corresponding to different transmission environments and network sizes. Stemming on the results of the analysis, in Table 4, we provide a synopsis highlighting the most energy efficient architecture in the above-mentioned scenarios.

Finally, it is worth noting that, even when not preferable from the point of view of consumed energy, in-network reconstruction may still be preferable since it can provide bandwidth saving. This occurs when the fraction of measurements and the CS measurements quantization accuracy needed for accurate reconstruction are large w.r.t. the cost of conventional encoding.

## 6. Adoption of Energy Efficient CS Reconstruction Algorithms for IoT Networks

The CS reconstruction problem is typically ill-posed and affected by noise, and reconstruction algorithms’ performances are typically compared in terms of accuracy as well as computational complexity. In this section, we show how, given our analysis of energetic implications of the reconstruction stage, reconstruction algorithms can be compared in terms of consumed energy as well.

**Figure 7:**
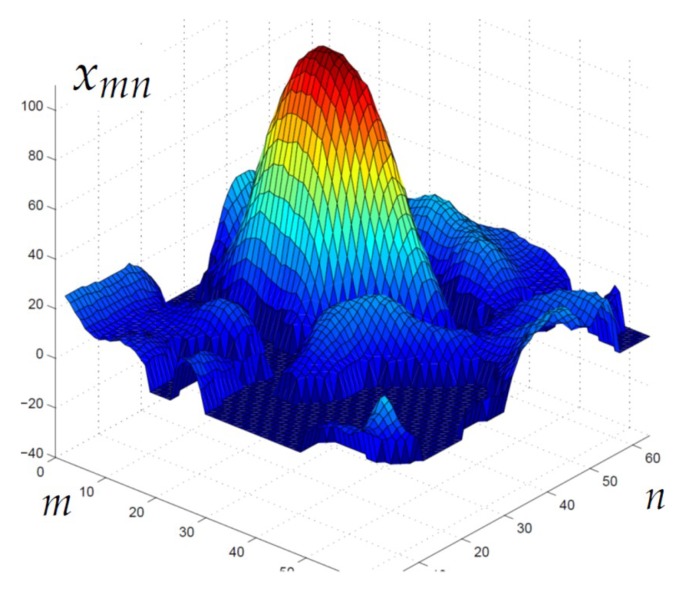
Oceanographic field (N=64×64 ).

Specifically, we here use the above analysis to compare different CS reconstruction algorithms to be used in IoT networks, in terms of energy vs. accuracy trade-off. We consider the oceanographic image depicted in Figure 7, which is a 64×64 detail of an oceanographic field image from the database in [30]. Without loss of generality, the CS acquisition is carried out using an M×N Gaussian matrix.

We analyze the energy and accuracy for the reconstruction algorithm CoSaMP [31], the algorithms [32,33], and a modified version of CoSaMP referred to as Nonlinear CoSaMP (NLCoSaMP). The NLCoSaMP algorithm, introduced in [34], leverages Bayesian priors within a CoSaMP-like iterative structure. The rationale behind NLCoSaMP is that, when a prior assumption is available for the vector α in Equation (1), the best minimum mean square error estimate of α given a noisy estimate $\hat{\alpha }$ can be computed by the conditional expectation $E\{\alpha |\hat{\alpha }\}$. Thereby, the signal estimate at the generic iteration of the CoSaMP is improved by a soft-thresholding stage implementing the conditional expectation above, and the solution drifts towards satisfying both the CS measurements and the prior. This key idea, formerly applied to CS reconstruction of astronomical images [35], leads to different nonlinear algorithms depending on the actual prior. In the light of the work in [34], we consider here a normal prior on wavelet coefficients’ trees such that, at the generic iteration, each wavelet coefficient tree obtained by CS measurements pseudo-inversion is replaced with its nonlinearly estimated counterpart. NLCoSaMP has the same computational complexity of as CoSaMP, and it is well suited to in-network reconstruction.

**Figure 8:**
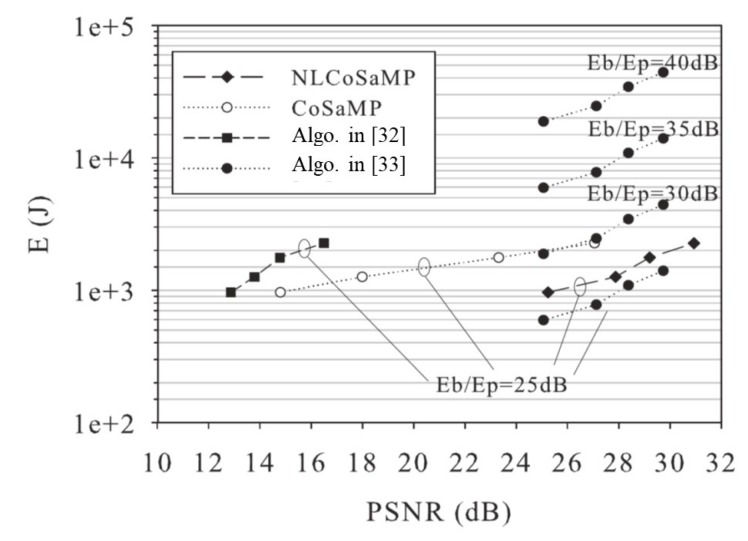
Energy E(J) vs. PSNR for in-network CS reconstruction algorithms (NLCoSaMP, CoSaMP, and [32]) and off-network one ([33]).

Figure 8 plots the overall reconstruction energy (in Joule) vs. the achieved accuracy, measured in terms of Peak Signal-to-Noise Ratio (PSNR: The PSNR is defined as $\mathrm{PSNR}=255^{2}/\mathrm{MSE}$ where MSE represents the Mean Square value of the reconstruction Error.)

NLCoSaMP, CoSaMP, and the algorithm [32] are well suited to perform in-network reconstruction, and their energy is computed under this condition, i.e., $E\left(J\right)=E_{\mathrm{in}-\mathrm{net}}$ in accordance with Equation (4). The algorithm in [33], being computationally heavy, is assumed to perform off-network reconstruction, and the energy spent by the network in this case (i.e., the energy needed to transmit the CS measurements out of the network) is computed as $E\left(J\right)=E_{\mathrm{of}-\mathrm{net}}$ in accordance to Equation (3).

We have set *E*${}_{p}$ = 0.15 μJ (see [25]) and we have compared the algorithms under different values of the ratio $E_{b}/E_{p}$ (dB). The analysis allows to compare the algorithms in terms of energy versus accuracy trade-off. Remarkably, the adoption of Bayesian prior in NLCoSaMP translates into an energy efficiency improvement w.r.t. the CoSaMP algorithm; the NLCoSaMP algorithm systematically requires less energy with respect to all the competitors for in-network reconstruction and with respect to the algorithm in [33] (apart the case of very low $E_{b}/E_{p}$ (dB), that may correspond to low transmission rate or high-quality channel). It is worth observing that the plot accounts for in-network energy only and disregards the reconstruction energy of the algorithm in [33], which is spent out of the IoT network and not relevant in this comparison. In the light of these results, we remark that computationally feasible algorithms may achieve the same accuracy as more complex one. Nonetheless, their superior energy efficiency may motivate their adoption for CS reconstruction in the typically energy constrained environment of IoT networks. Let us finally observe that the herein reported performance comparison, based on the methods in [32,33] and their numerical implementation in [36,37], may serve as a paradigm for different classes of reconstruction algorithms. In fact, several CS algorithms such as [38,39,40] can be categorized in accordance with their algorithmic structure, computational complexity and performances, as illustrated in depth in [41]. Thereby, joint evaluation of the selected accuracy metric and of the energy consumption estimated according to Equations (3) and (4) can lead to the selection of the algorithm best matching the particular IoT scenario under concern.

## 7. Conclusions

In this work, we have presented a novel analysis aimed at identifying green CS reconstruction computing architectures and energy efficient CS reconstruction algorithms to be used in IoT networks for environmental monitoring. The analysis computes the energy consumption within the IoT network under two computing architectures, where either CS measurements are forwarded to off-network devices for reconstruction and storage or CS reconstruction takes place within the IoT network and the reconstructed data are encoded and transmitted out of the IoT network. Our analysis allows for comparing the two architectures in terms of consumed energy. We show how to exploit the analysis to identify the most energy efficient architecture in scenarios differing in network size as well as transmission technologies. As a further novel contribution, we present a performance comparison of different CS reconstruction algorithms by taking into account the consumed energy, and we analyze the energy vs. accuracy trade-off. The presented approach highlights efficient CS reconstruction schemes to be adopted in green CS architectures for IoT networks.

References

## Figures and Tables

**Table 1:** Notation.

Parameter	Value
*T*	Observation time
$T_{s}$	Temporal sampling interval
*M*	Number of CS measurements per snapshot
*L*	Bits per CS sample
*N*	Size of the sampled field
$\rho_{CS}$	CS compression ratio M/N
$\rho_{ENC}$	Video codec compression ratio
$E_{b}$	Per bit transmission energy
$E_{p}$	Per elementary operation processing energy
$E_{\mathrm{off}-\mathrm{net}},E_{\mathrm{in}-\mathrm{net}}$	IoT network energy consumed during *T* (Off-network, In-network reconstruction)
$B_{\mathrm{off}-\mathrm{net}},B_{\mathrm{in}-\mathrm{net}}$	Bandwidth required at the sink output (Off-network, In-network reconstruction)
$R_{in}$	Ratio of the energy costs of the two strategies $\frac{E_{\mathrm{in}-\mathrm{net}}}{E_{\mathrm{off}-\mathrm{net}}}$

**Table 2:** In-network bandwidth savings versus CS to video codec relative compression efficiency.

Relative Compression Efficiency [dB]	In-Network Bandwidth Saving
$\rho_{\mathrm{CS}}/\rho_{\mathrm{enc}}$ [dB]	$B_{\mathrm{in}-\mathrm{net}}/B_{\mathrm{off}-\mathrm{net}}$
0	12.5%
5	3.9%
10	1.2%

**Table 3:** Maximum field size $N_{in}$ s.t. $D_{in}=1$ for different IoT network technologies.

Scenario	Device	$E_{b}/E_{p}$	$N_{\mathrm{in}}$
Wireless	IEEE 802.15.4 compliant	20 dB	≈800
Underwater	Acoustic modem [26]	50 dB	≈$10^{5}$
Underwater	Acoustic modem [27]	70 dB	≈$10^{7}$

**Table 4:** Most efficient architecture for different IoT network scenarios ($\rho_{CS}/\rho_{enc}=20\;\mathrm{dB}$, L=8).

Scenario	$N\approx 10^{3}$	$N\approx 10^{4}$	$N\approx 10^{5}$
Wireless ($E_{b}/E_{p}\approx 20\;\mathrm{dB}$ )	In-Network	Off-Network	Off-Network
Underwater, Low power ($E_{b}/E_{p}\approx 50\;\mathrm{dB}$ )	In-Network	In-Network	Off-Network
Underwater, High power ($E_{b}/E_{p}\approx 70\;\mathrm{dB}$ )	In-Network	In-Network	In-Network
